# Bioinstructive polymer fibre mats to reduce bacterial pathogen colonisation

**DOI:** 10.1039/d5lp00220f

**Published:** 2025-11-17

**Authors:** Joseph Sefton, Michael P. Avery, Jean-Frédéric Dubern, Mohammad Ghasemzadeh-Hasankolaei, Rahul Tiwari, Amir M. Ghaemmaghami, Morgan R. Alexander, Paul Williams, Derek J. Irvine, Jonny J. Blaker, Adam A. Dundas

**Affiliations:** a Department of Chemical and Environmental Engineering, Faculty of Engineering, University of Nottingham Nottingham NG7 2RD UK adam.dundas1@nottingham.ac.uk; b Henry Royce Institute, The University of Manchester Manchester M13 9PL UK; c National Biofilms Innovation Centre, Biodiscovery Institute and School of Life Sciences, University of Nottingham NG7 2RD UK; d School of Life Sciences, University of Nottingham NG7 2RD UK; e Camstent Ltd, The Exchange Colworth Science Park Bedford MK44 1LZ UK; f Advanced Materials & Healthcare Technologies, School of Pharmacy, University of Nottingham NG7 2RD UK; g Centre for Additive Manufacturing, The University of Nottingham Nottingham NG7 2RD UK; h Department of Materials, The University of Manchester Manchester M13 9PL UK

## Abstract

Healthcare associated infections are widely reported to cost the European economy alone over £20 billion per year and cause an estimated extra 25 million hospital days considerably increasing patient morbidity and mortality. Implanted medical devices have previously been developed without the consideration of their potential to harbour pathogens at their surface, and this has resulted in many devices that suffer from bacterial biofilm colonisation and fibrotic foreign body responses that cause inflammation and inhibit wound healing. Here we report the development of a fibrous bioinstructive co-polymer mat that reduces biofilm formation by *Pseudomonas aeruginosa* and *Staphylococcus aureus* by 84% and 59% respectively compared to poly(lactic acid) fibres. The fibres also promote proliferation of fibroblast cells by 2.2-fold over 3 days compared to 1.2-fold for poly(lactic acid) samples, showing that the fibres promote a wound healing environment. Through the development of new materials for bioinstructive meshes, this work aims to develop new materials that can be used for surgical meshes that can prevent infections without the need for antimicrobials or toxic leaching compounds.

## Abstract 2

Development of a bioinstructive polymer mat using electrospinning that prevents biofilm formation and promotes fibroblast proliferation through surface chemistry interactions.

## Graphical abstract

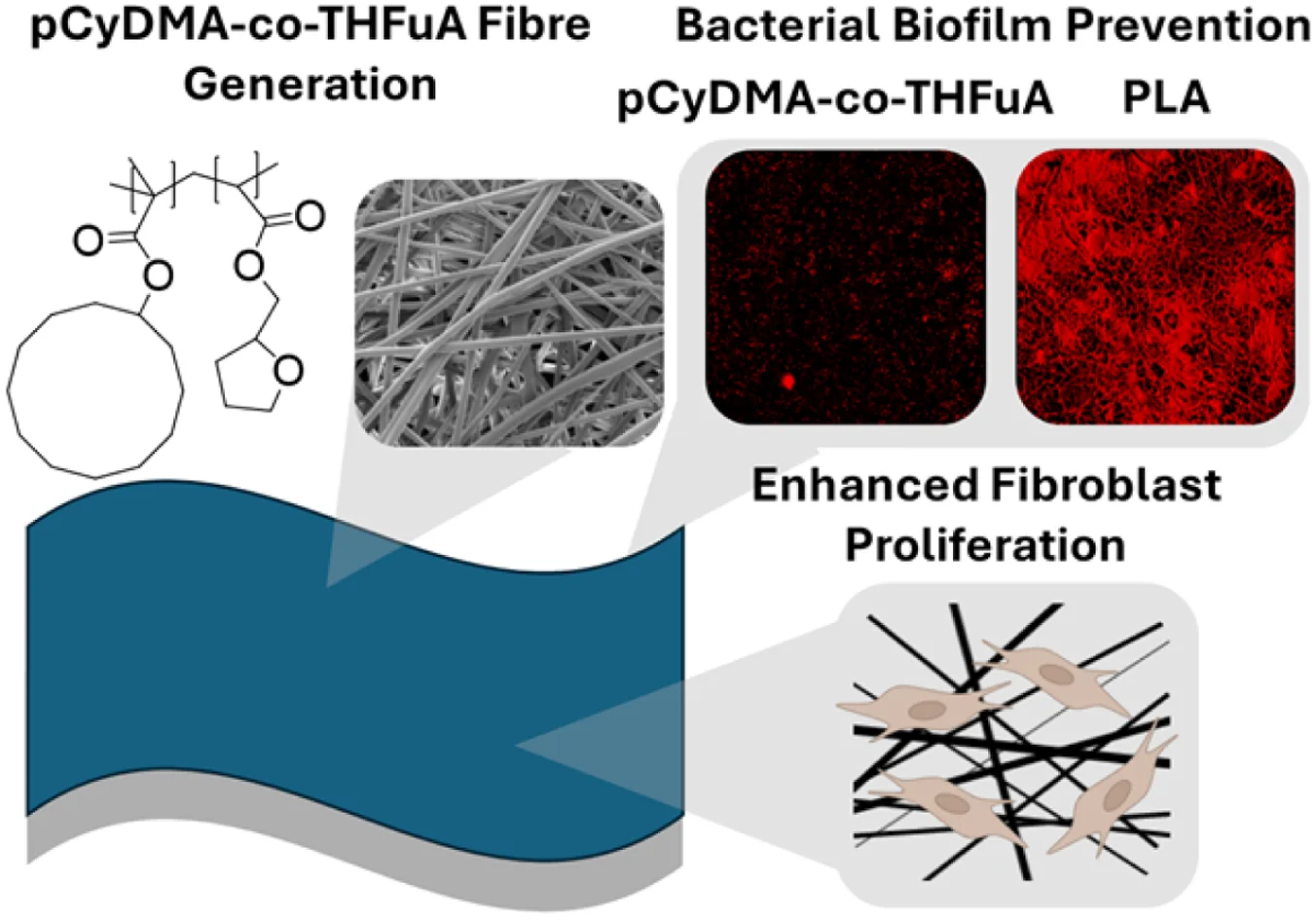

## Introduction

Surgical site infections (SSIs) are among the most common healthcare associated infection. SSIs are associated with increased morbidity and mortality as well as costly prolonged hospital stays.^1^ The use of surgical meshes is a standard procedure in hernia surgery and reduces recurrence rates by up to 64% when compared to stitching.^2^ However, infection rates are between 1–8% depending on the surgical procedure.^3^ Many new meshes that claim to prevent infection have been approved for clinical use, typically using antimicrobial coatings to kill bacteria. Importantly, this mechanism applies selective pressure to microbes, which can encourage antimicrobial resistance. The most common pathogens associated with hernia meshes are *Staphylococcus aureus* (including methicillin *S. aureus* resistant (MRSA)) and *Staphylococcus epidermidis*.^4^ Common Gram-negative bacteria associated with mesh infections include *Pseudomonas aeruginosa*, *Escherichia coli* and *Klebsiella spp*.^5^ In clinical practice, surgical meshes have also been shown to cause inflammation, which can be due to infection, but has also been linked to the foreign body reaction leading to a slower healing process post-surgery.^6^ A new bio-instructive surgical mesh that can prevent bacterial biofilm formation without the incorporation of antimicrobials as well as reducing inflammation would represent a significant step forward in hernia repair.

Surgical meshes are typically woven, which provides increased strength arising from interlaced fibres. This is particularly important for devices such as hernia meshes that support damaged tissues during wound healing.^7^ An emerging manufacturing technique for creating hernia meshes is electrospinning. Electrospinning can form nano-to micro scale diameter fibres in non-woven mats in a continuous, scalable and cost-effective process, where the fibre morphology can be fully controlled by formulation and the spinning parameters chosen. Spinning onto a flat plate collector typically produces non-woven mats with fibres in random orientations. These mats have several advantageous properties for use in wound healing and in hernia repair.^8,9^ The entanglement and slight merging of fibres at points of contact results in the formation of a structurally sound and self-supporting mat which remains functional even when cut. Fibre diameters in the nanometre to micrometre range, along with a high surface to volume ratio and good porosity which, as a result, can mimic the structure of the extracellular matrix (ECM).^10^ These properties provide many sites for cell adhesion and proliferation whilst maintaining access for nutrients and gas exchange *via* the interconnected porous network.^11^ The high surface area of the fibres has the additional advantage of improving the efficacy of any surface-active properties of the fibre material or any active ingredient fillers.

A new class of bio-instructive polymers has previously been discovered with properties that can prevent both bacterial and fungal biofilm formation and promote stromal cell proliferation and immune homeostasis.^12–14^ Poly(cyclododecyl methacrylate) (pCyDMA) was previously shown to prevent biofilm formation of six clinically relevant bacterial pathogens and has been proposed as a coating for urinary catheters to prevent catheter-associated urinary tract infections.^15^ The mechanism of action, whilst still being elucidated, suggests that rigid (meth)acrylates that are more hydrophobic are able to prevent bacterial biofilm formation more successfully.^16^ Another polymer made from tetrahydrofurfuryl acrylate (THFuA) has been used to create functional microparticles capable of increasing attachment and proliferation of fibroblast cells, which were then shown to accelerate healing in a diabetic mouse wound model.^17^ A significant issue with these two materials is their mechanical properties where pCyDMA is very brittle and pTHFuA is very soft, making the realisation of a medical device entirely from these individual materials difficult.

**Fig. 1:**
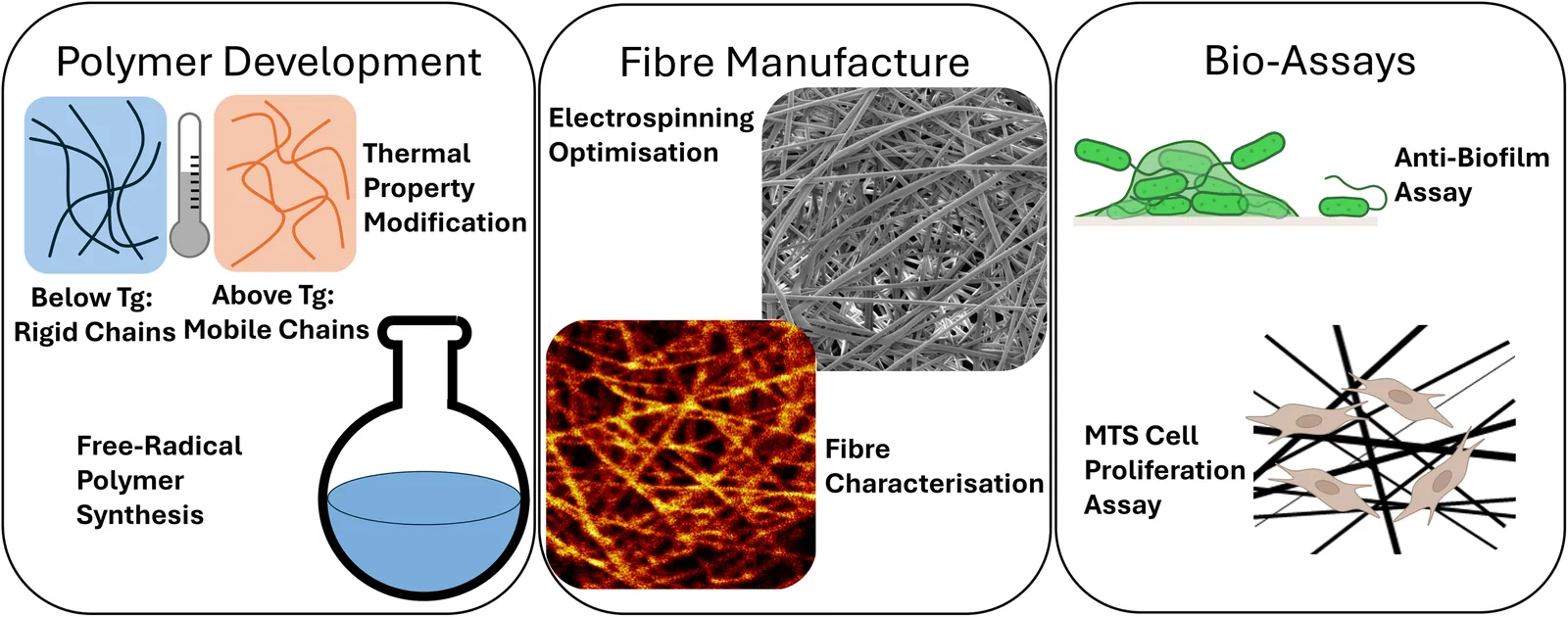
Overview figure showing the scheme of work showing (a) Polymer development work by using two monomers to create a polymer with tunable thermal properties. (b) Optimisation of electrospinning process to develop scalable polymer mats with characterisation from techniques including SEM and ToF-SIMS. (c) Polymer mats were placed into two different biological assays, a single species anti-biofilm assay and an MTS cell proliferation assay to demonstrate the fibres ability to prevent biofilm formation and promote proliferation of fibroblast cells.

Here, we report the synthesis of a co-polymer, pCyDMA-*co*-THFuA, that has optimised thermal properties to give a glass transition temperature of 29.2 °C, making it suitable for processing by electrospinning into fibres and enable easier handling for the constructed mat. This candidate material has also been shown to reduce bacterial biofilm formation and to promote accelerated wound healing (Fig. 1). This new bioinstructive polymer was shown to be capable of being processed using electrospinning to form polymer fibres of a diameter of 2.28 ± 0.65 µm. Large area (20 cm × 25 cm) fibre mats were produced to demonstrate both scalability and to produce samples for biological assays. The pCyDMA-*co*-THFuA fibres were able to resist both *P. aeruginosa* and *S. aureus* biofilm formation. Fibroblast attachment and proliferation was also supported during 3 days of cell culture.

## Experimental

### Polymer synthesis

Cyclododecanol (>99.5%), tetrahydrofurfuryl acrylate (THFuA, >99%), butyl methacrylate (>99%), THFuA (>80%), Ti(OBu)_4_ (97%) and azobisisobutyronitrile (AIBN) (98%) were obtained from Sigma-Aldrich. Toluene (99%), methanol (99%) and dichloromethane (99.8%) were obtained from Fisher. Dichloromethane (≥99%) was obtained from Sigma-Aldrich (Merck). Ethanol abs. (≥99.8%) was obtained from VWR. CyDMA was synthesized as previously described *via* transesterification of butyl methacrylate with cyclododecanol,^18^ and purified *via* vacuum distillation to remove residual BuMA, then passed over a plug of silica to remove the majority of residual Ti(OBu)_4_. The purity of CyDMA was confirmed by ^1^H NMR spectroscopy.

Polymers were prepared at a scale of 25 g in toluene (2 : 1 v/w solvent : monomer) by free-radical copolymerization of CyDMA and THFuA using AIBN (0.5 mol%) as the radical initiator. The reactions were carried out as batch reactions. Reaction solutions were sparged with N_2_ gas for 1 h with an inlet for N_2_ gas and an outlet to equalise pressure and allow for the removal of oxygen and then sealed under N_2_ prior to heating at 80 °C for 4 h. Once 4 h had elapsed, solutions were cooled, and crude samples were analysed by ^1^H NMR to determine conversion. Polymers were precipitated by first precipitating the crude solution into a 20-fold excess of cold methanol, redissolving the precipitate to approximately 50 mg mL^−1^ in DCM and precipitating dropwise into a 10-fold excess of cold methanol twice more. The precipitate was isolated by vacuum filtration and dried over air for *ca*. 1 h before being dried in a vacuum oven at 25 °C for 2 days in a vacuum oven, then crushed into a fine powder and further dried *in vacuo* for 5 days. The dried products were analysed by ^1^H NMR to determine comonomer ratio compared to the theoretical starting ratio, and GPC to determine molecular weight and dispersity. Prior to use for cell culture, 2D polymer samples were prepared by dip-coating 13 mm round glass coverslips into a 10% w/v polymer solution in DCM.

### Proton nuclear magnetic resonance (^1^H NMR) spectroscopy

^1^H NMR spectra were recorded as the average of 16 scans at approximately 10 mg mL^−1^ in CDCl_3_ at 400 MHz on an AV400 spectrometer (Bruker Inc., Germany). Chemical shifts were recorded in *δ*_H_ referenced against the residual CHCl_3_ signal (7.26 ppm) and were processed using TopSpin 4.1.4 software (Bruker Inc., Germany). ^1^H NMR was utilized to determine conversion of monomer to polymer by comparing monomeric and polymeric resonances.

### Gel permeation chromatography (GPC)

Polymer samples were dissolved at 2 mg mL^−1^ in HPLC-grade THF, filtered through 0.22 μm PTFE syringe filters and analysed by GPC at a flow rate of 1 mL min^−1^ at 35 °C through a 7.5 × 50 mm PLGel MIXED guard column (5 μm particle size) and two 7.5 × 300 mm PLGel Mixed-C columns (Agilent Technologies, Inc., CA, USA) in series to a differential refractive index detector (Agilent Technologies, Inc., CA, USA). Calibration was achieved using 12 narrow dispersity PMMA standards in the range of 540–2 210 000 g mol^−1^ (Agilent Technologies, Inc., CA, USA). GPC chromatograms were analyzed using the Astra 6.1.7 software package (Wyatt Technology Corp., CA, USA). GPC was utilized to determine *M*_n_, *M*_w_, and *Đ*.

### Differential scanning calorimetry

DSC was performed on *ca.* 4 mg of polymer using a Q2000 instrument (TA Instruments). Ramp rates rate of 5 °C min^−1^ were utilized between 0–200 °C for pCyDMA homopolymer and pCyDMA-*co*-THFuA 75 : 25, and between −20–150 °C for pCyDMA-*co*-THFuA 50 : 50. A heating cycle was first utilized to erase any thermal history in the sample, followed by a cooling cycle and a second heating cycle. The *T*_g_ was taken as the midpoint of the inflection in the heat flow of the second heating cycle. Analysis was performed using TA Universal Analysis software.

### Electrospinning

Solutions for electrospinning were prepared by dissolving the CyDMA polymer or CyDMA-*co*-THFuA copolymer in DCM with stirring at room temperature in the required w/v ratio. For dual solvent systems, ethanol was then added to this DCM/polymer stock solution to give the final required DCM : EtOH ratio and polymer w/v. Addition of EtOH caused the polymer to precipitate but would then redissolve with further stirring. To produce PLA fibres, polylactide PLA3051D (*M*_w_ = 93 500 g mol^−1^, 96% l-lactide, 4% d-lactide) manufactured by NatureWorks LLC (USA) was dissolved in DCM with stirring at room temperature. For dual solvent systems, ethanol was then added to this DCM/polymer stock solution to give the final required DCM : EtOH ratio and polymer w/v.

Electrospinning of the polymer solutions was conducted using a Fluidnatek LE-500 pilot-scale electrospinning platform with built-in environmental control, Bioinicia S.L. (Valencia, Spain). The system can independently provide ±30 kV to both emitter and collector. It can operate in laboratory mode with a single emitter for experimental design, with the capability for scale up *via* high throughput multi nozzle emitters and a roll-to-roll collector. To allow for small volume spinning and optimisation of spinning conditions, spinning was conducted using a single emitter setup using a 20G (0.9 × 0.6 mm OD × ID) flat nosed capillary needle with a flat plate collector at a working distance of 24 cm. Fibres were spun onto aluminium foil (matt side), to provide a support compatible for further chemical and biological testing, and non-stick baking paper (Sainsburys, UK), to allow for easy removal of self-supporting fibre mats. Fibres on the foil substrate were used for further biological testing to ensure samples remained in a flat, fixed position for testing. Environmental controls in the chamber were set to 20 °C, 40% RH and air flow of 90 m^3^ h^−1^.

### SEM determination of fibre morphology and size fibres

Samples were mounted onto aluminium sample stubs using double sided carbon pads and coated in a 10 nm layer of Au : Pd 80 : 20 using a Quorum Q150T ES plus sputter coater (Quorum Technologies Ltd UK) prior to imaging by scanning electron microscopy. Electron micrographs were collected using a TESCAN VEGA 3 Scanning Electron Microscope (TESCAN, Brno, Czech Republic) using a 5 kV accelerating voltage. Average fibre diameters (AFDs) and standard deviation were calculated from measuring 100 fibres at a suitable magnification using ImageJ.

### Surface chemistry analysis by time of flight-secondary ion mass spectrometry (ToF-SIMS)

A ToF-SIMS V (IONTOF GmbH) instrument using a 25 keV Bi_3_^+^ primary ion source was used for the bulk of the analysis. Bi_3_^+^ primary ions were used with a target current of ∼0.3 pA. Analysis for positive and negative spectra was acquired over a 500 µm × 500 µm scan area. Other analyses parameters were a cycle time of 100 µs, one shot/frame/pixel, one frame/patch and 20 scans per analysis. As the samples were of a non-conductive nature, charge compensation in the form of a low energy (20 eV) electron flood gun was applied. Images and spectra were acquired using SurfaceLab 7 software and analysed using SurfaceLab 7.1 software.

### Preparation of 2D polymer samples

To prepare flat polymer samples, the desired polymers were dissolved in DCM at 10% w/v. Clean tweezers were used to hold 13 mm circular glass coverslips at the edge, and one face of the coverslip was contacted with the solution. The coverslip was then removed and excess solvent allowed to evaporate before the cover slip was placed into a Petri dish. Further solvent evaporation was allowed to occur under gentle airflow in a fume cupboard for a further 72 h before use.

### Microbiological assays

For biofilm quantification, samples were placed in 6-well microplates (Corning). *P. aeruginosa* strain PAO1 (Washington subline) and *S. aureus* strain SH1000, were transformed respectively with plasmids pMMR and pBKminiTn7-*egfp* that constitutively express either mCherry or green fluorescent protein (GFP).^19,20^ The fluorescently tagged strains were grown at 37 °C for 16 h in RPMI-1640 (Lonza, Slough, UK), diluted 1 : 100 (v/v) in fresh medium and allowed to grow until an optical density at 600 nm (OD_600_) of 0.5 was reached. These cultures were diluted to OD_600_ 0.1 for *S. aureus* and 0.01 for *P. aeruginosa* in RPMI-1640 and inoculated into 6 well microplates containing UV sterilised samples. Bacterial cell cultures were incubated with shaking for 48 h for *S. aureus* or 24 h for *P. aeruginosa* to form mature biofilms. Samples were gently washed twice in phosphate buffered saline (PBS) and once in de-ionized (DI) water to remove any loosely adhered bacteria. Samples were then examined using confocal laser scanning microscopy (Zeiss LSM700, Zeiss, Oberkochen, Germany) using eGFP and mCherry modes at excitation wavelengths of 488 nm and 555 nm respectively. Imaging was conducted using Zen 2011 imaging software (Zeiss, Oberkochen, Germany). A total of 5 Z-stacked images were collected per sample. Sampling was conducted at random from the central portion of each sample. Biomass was calculated using Image J (NIH, Bethesda, MD, USA) and the Comstat 2.1. Software package (https://www.comstat.dk, Lyngby, Denmark) was used to apply intensity thresholds to distinguish biomass from background. Biovolume (µm^3^ µm^−2^) was computed by summing the volume of all voxels identified as biomass across the stack and normalizing to the substratum area.^21^

### Cell culture

To assess the biocompatibility of the synthesized polymers, BJ6 fibroblasts were cultured on the polymer samples as an example cell line that represent a well-characterised, skin-derived fibroblast model widely used in investigating wound-healing processes.^22^ Passage 7 BJ6 fibroblasts (ATTC) were cultured in a T75 flask using DMEM-GlutaMax (Gibco, Thermo Fisher Scientific) supplemented with 10% foetal bovine serum (FBS; Sigma-Aldrich) and 1% penicillin/streptomycin (Sigma-Aldrich) in a CO_2_ incubator at 37 °C. Once the cells reached approximately 90% confluency, they were harvested using 0.025% trypsin/EDTA (Sigma-Aldrich), and a cell suspension with a density of 2 × 10^6^ cells per mL was prepared. Coverslips coated with various polymers (2D: pCyDMA, pTHFuA and pCyDMA-*co*-THFuA) and fibre mat samples (PLA and pCyDMA-*co*-THFuA) were first UV-sterilized twice (20 minutes each time) using a UVClave machine (Benchmark, USA), which is kept in a class II cell culture hood, and then placed into wells of non-tissue culture-treated 24-well plates (8 wells per polymer, with one 2D or fibre mat sample per well). This enabled replicates of *n* = 4 for each of Day 1 and Day 3 testing. Then, 50 µL of the cell suspension containing 1 × 10^5^ BJ6 cells was seeded onto each polymer. The plates were incubated at 37 °C for 3 h to allow for initial cell attachment. Following incubation, all samples were gently washed with culture medium to remove any unattached cells, then transferred to fresh non-tissue culture-treated 24-well plates. Each well was then topped up with 1 mL of culture medium and cultures continued for up to 3 days.

### MTS proliferation assay

To assess the effects of different polymer substrates on fibroblast viability and proliferation, an MTS assay was performed on the cultured cells using the Abcam colorimetric MTS assay kit (ab197010) on days 1 and 3 post-culture (*n* = 4 per polymer per assay day). For the assay, all samples were washed twice with PBS and transferred to fresh 24-well plates containing 210 µL of PBS per well. Then, 100 µL of MTS reagent was added to each well, and the plates were incubated at 37 °C for 3 hours. Following incubation, three 100 µL aliquots from each well of the 24-well plate were transferred into three separate wells of a flat bottom 96-well plate and the optical density (OD) was measured at 490 nm using a plate reader (Thermo Fisher Scientific). A triplicate set of control wells without cells was used to determine and subtract background absorbance. To determine the number of cells on each platform on the assay days, the obtained OD values were compared to a standard curve (Fig. S1) generated previously from known densities of BJ6 cells using the MTS assay.

## Results and discussion

As poly(tetrahydrofurfuryl acrylate) (pTHFuA) has been shown to increase fibroblast proliferation,^17^ it was a highly desirable component when designing electrospun fibers for wound-healing applications. However, the *T*_g_ of p(THFuA) is sub-ambient (∼−13 °C), such that electrospinning as a homopolymer would have resulted in fibres conjoining and forming a film over time. Thus, a co-monomer was required to yield a copolymer with an above-ambient *T*_g_. CyDMA forms a homopolymer with *T*_g_ ≈ 88 °C and has previously been shown to have anti-biofilm properties, which would also be of benefit in surgical meshes. This makes it an ideal candidate for tuning the *T*_g_ of a functional co-polymer series that is processable *via* electrospinning and remains a solid at body temperature.

**Fig. 2:**
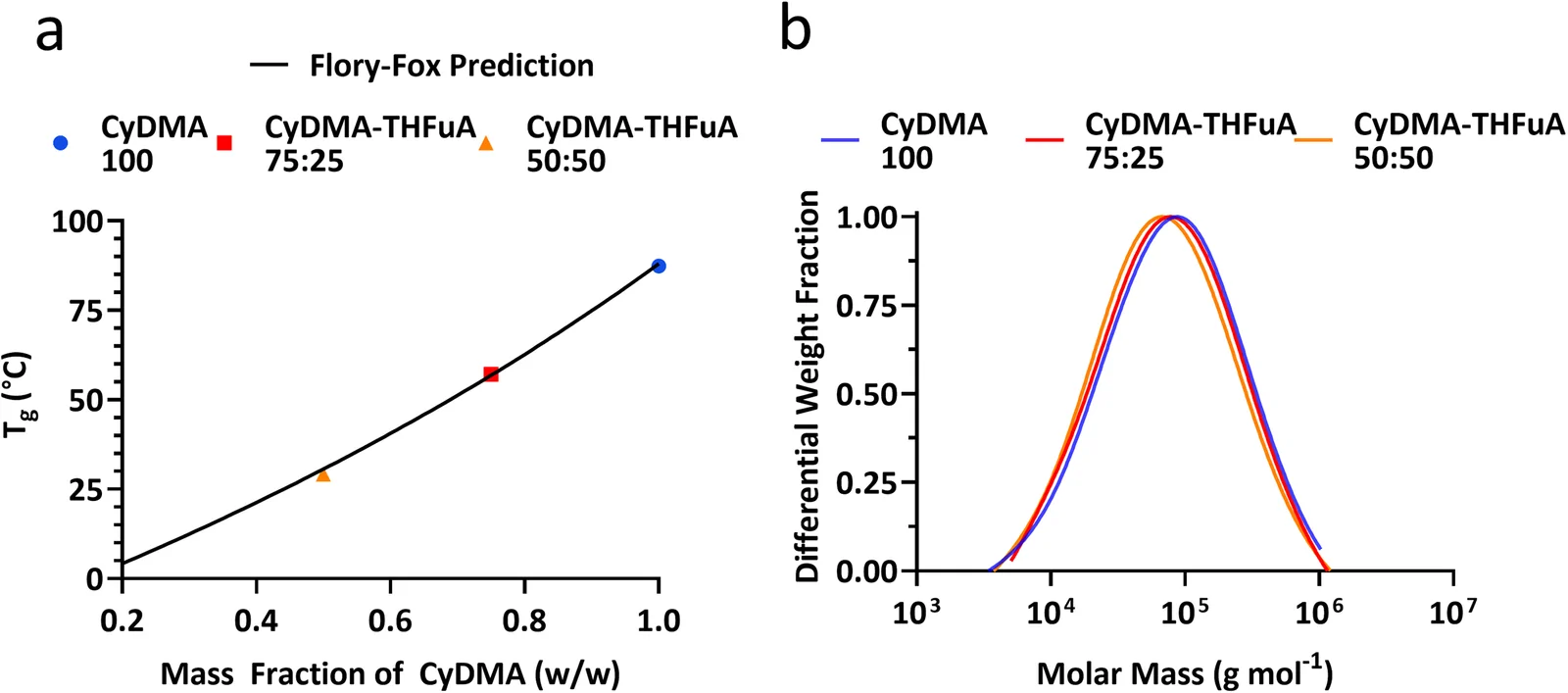
Thermal and physical analysis of the synthesised polymers. (a) The *T*_g_ trend predicted by the Flory-Fox equation (black line) and experimentally determined *T*_g_s for CyDMA-*co*-THFuA copolymers, (b) molecular weight distributions obtained by GPC. Blue – pCyDMA homopolymer, red – CyDMA-*co*-THFuA (75 : 25) and Orange – CyDMA-*co*-THFuA (50 : 50).

CyDMA homopolymer and two CyDMA-*co*-THFuA (75 : 25 and 50 : 50 w/w) copolymers were synthesized using free-radical polymerisation, targeting *T*_g_s of 30 to 88 °C, as predicted by the Fox equation.^23^ Experimentally determined polymer *T*_g_s ranged from 29 to 87 °C when measured by DSC (Fig. S2), in agreement with the Fox equation prediction (Fig. 2a). Molar calculation of the feed and product copolymer ratios showed that CyDMA and THFuA were highly co-reactive with the product copolymer feed closely matching the feed ratio (≤0.5 mol%). Additionally, the synthesized polymers displayed similar *M*_w_ distributions by GPC analysis, as shown in Table 1 and graphically in Fig. 2b.

**Table 1:** Physical and thermal characterization of the polymers utilized in this study determined by GPC and DSC

CyDMA : THFuA^a^ (% w/w)	*M*_n_ b (g mol^−1^)	*M*_w_ b (g mol^−1^)	*Đ* b	*T*_g_ c (°C)
100 : 0	43 500	135 400	3.11	87.4
75 : 25	42 500	134 500	3.16	57.1
50 : 50	41 200	126 200	3.07	29.2

a Feed ratio.

b Determined by GPC analysis.

c Determined by DSC analysis.

To process the polymers into bio-instructive polymer mats, electrospinning was used to create non-woven fibre mats with a random fibre orientation. The three polymer compositions were first dissolved in DCM to test the electrospinning performance as a function of polymer composition and concentration. It was found that a polymer concentration of 10 w/v was insufficient to form fibres of any composition whilst a concentration of 30 w/v was prone to emitter clogging. Therefore, a concentration of 20 w/v was determined as optimal. Even with these optimised conditions, the homopolymer CyDMA was found to spin poorly and erratically with large solid deposits forming on the emitter and clogging of the emitter. The resulting fibres were sparse and of large diameter with ribbon-like morphology and beading. For the co-polymers, an increasing THFuA content was found to improve the spinnability of the solutions, reducing emitter clogging and improving the stability of the polymer jet. The fibres exhibited bead-on-a-string morphology which required further optimisation before good quality fibre mats could be fabricated.

Fibre morphology and spinning consistency were improved by using a dual-solvent system that was developed and tested on the most promising candidate material pCyDMA-*co*-THFuA (50 : 50). EtOH was added to DCM in increasing amounts to reduce the surface tension of the solvent system and improve the fibre morphology. Solvent systems comprising 1 : 0, 6.5 : 1, 4 : 1 and 2 : 1 DCM : EtOH were used and found to fully solvate the polymer, despite EtOH alone being a poor solvent for this material. As EtOH content increased, fibre morphology transitioned from beads on a string to smooth continuous fibres when using a 2 : 1 DCM : EtOH solvent system (Fig. S4). Further optimization of the spinning parameters of this 2 : 1 DCM : EtOH solvent system with pCyDMA-*co*-THFuA (50 : 50) at 20 w/v found the best balance between fibre morphology and consistent, stable spinning using a solution flow rate of 5 mL h^−1^ and −20/+1 kV emitter/collector.

**Fig. 3:**
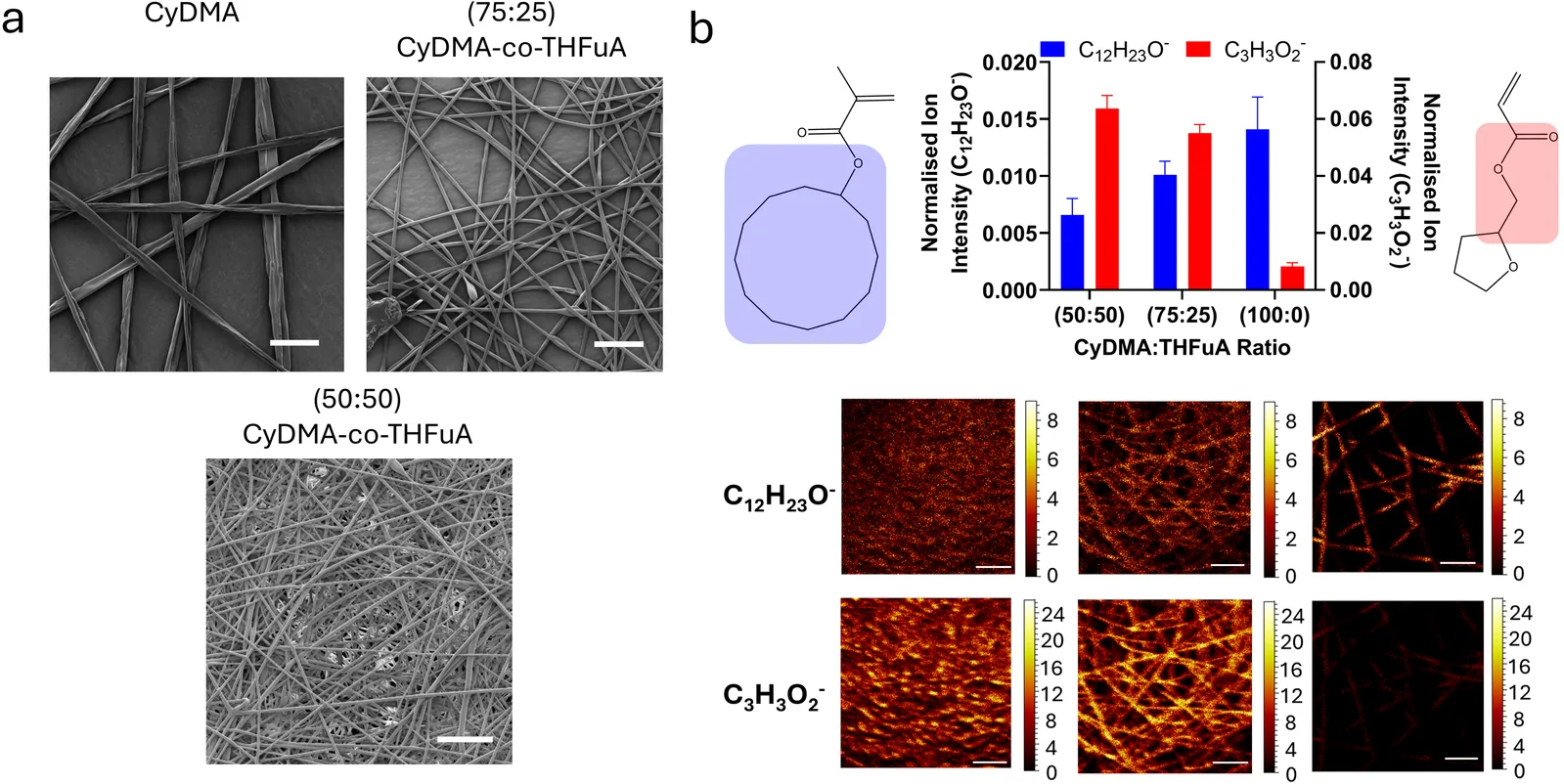
(a) Representative SEM images showing fibre structure for CyDMA-*co*-THFuA (50 : 50), CyDMA-*co*-THFuA (75 : 25) and CyDMA fibres. Scale bars = 50 µm. (b) ToF-SIMS data showing intensities of 2 key ions associated with 2 monomer structure (C_12_H_23_O^−^ = CyDMA, C_3_H_3_O_2_^−^ = THFuA) where the ions from the structures are circled in blue and red respectively. Samples (from left to right) include electrospun fibres containing: 50 : 50, 75 : 25 and 100 : 0 (CyDMA : THFuA). Ion images for C_12_H_23_O^−^ and C_3_H_3_O_2_^−^ – directly correlate to sample on the graph. Error bars shown represent ±1SD from *n* = 3 measurements.

To obtain thicker polymer mats that can be used in cell studies, a larger scale sample was made from pCyDMA-*co*-THFuA (50 : 50), using the optimized solvent and spinning conditions described above, in combination with the emitter translation capabilities of the equipment; with the emitter traversing in line back and forth, whilst maintaining the distance to the collector. Where previously samples had been produced in patch tests using short bursts of spinning in one location, now the emitter head was traversed back and forth across the substrate at a constant rate (50 mm s^−1^) and displacement (300 mm). A much larger area of the substrate could be covered with fibres using this technique, and a thick layer of fibres could be built up to form a self-supporting mat. This allowed for the manufacture of A4 sized sheets of non-woven fibrous material with multiple fibre layers with an average fibre diameter of 2.28 ± 0.65 µm to be formed (Fig. 3a). Fibres spun onto baking paper substrates could be removed easily as self-supporting mats whilst those on foil substrates adhered more strongly and were left fixed to the surface for further testing.

To be able to prevent biofilm formation and promote wound healing, cells need to be able to interact with the functional surface chemistry of manufactured fibre mats. Therefore, to confirm surface functionality, time of flight-secondary ion mass spectrometry (ToF-SIMS) was used to observe the surface chemistry. As the (75 : 25) CyDMA-*co*-THFuA and CyDMA polymers were not readily being processable *via* electrospinning, minimal fibres were manufactured as shown in Fig. 3a. However, these were able to be imaged by ToF-SIMS to demonstrate the relative change in signal peak intensity between the polymers. A unique ion (C_3_H_3_O_2_^−^) was identified for the pTHFuA component whilst C_12_H_23_O^−^ was identified to represent the cyclic hydrocarbon ring structure in pCyDMA. As expected, it was observed that a change in polymer ratio resulted in a shift in signal intensities, demonstrating that polymers with both CyDMA and THFuA components both presented these functionalities on the surface of the polymer (Fig. 3).

As these samples are targeting biomedical applications, a sterilization test was performed where the optimized pCyDMA-*co*-THFuA (50 : 50) sample was exposed to gamma radiation between 28.00 and 32.89 kGy (Swann Mortan, UK). The use of gamma radiation for sterilization is a standard procedure used for implanted medical devices. This can cause embrittlement of polymers,^24^ but no visible damage was observed to the fibres by SEM (Fig. S5).

To assess whether these surface functionalities were able to influence cellular behaviour, polymer fibres were initially exposed to *P. aeruginosa* and *S. aureus* as examples of relevant Gram-negative and Gram-positive bacterial species that are commonly responsible for chronic wound and surgical site infections.^25^ To compare bacterial biofilm formation on different fibre samples, an electrospun fibre made from PLA with comparable fibre diameter (1.20 ± 0.39 µm) was manufactured and assessed (Fig. S6). PLA meshes have been used surgically as an example of a resorbable mesh alternative.^26^ Equivalent 2D flat controls were included to observe the change in bacterial attachment between flat and fibre samples. These samples were made by making a solution of polymer into which glass coverslips were dipped.

**Fig. 4:**
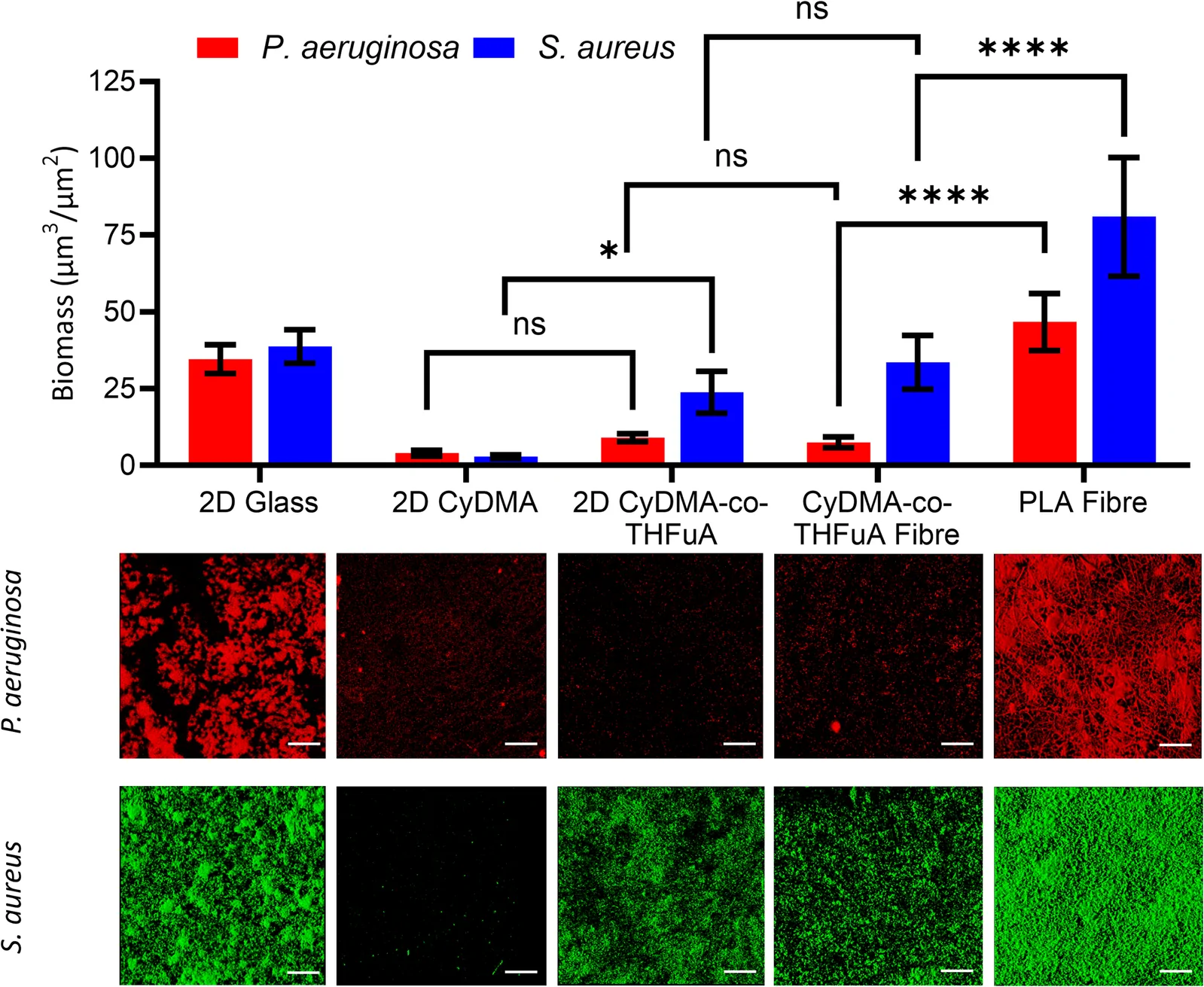
Surface coverage by single species (*P. aeruginosa* and *S. aureus*) biofilms quantified after 24 h incubation on glass, 2D CyDMA, 2D CyDMA-*co*-THFuA, PLA Fibre and CyDMA-*co*-THFuA Fibre. Error bars equal ±1 SD, *n* = 5. Statistical significance was calculated using one-way ANOVA where *p*-values * < 0.05, ** < 0.01, *** < 0.001 and **** < 0.0001 were used. The corresponding confocal microscopy images for mCherry labelled *P. aeruginosa* and egfp labelled *S. aureus*. Scale bars represent 100 µm.

Considering first the fibre samples, the biomass of both *P. aeruginosa* and *S. aureus* is significantly reduced on the pCyDMA-*co*-THFuA fibres compared to the PLA fibres by 84% and 59% respectively (Fig. 4). This demonstrates the ability of this material to effectively control pathogen biofilm formation. Comparing the functional fibres to 2D samples, *P. aeruginosa* showed no significant difference between the flat 2D pCyDMA-*co*-THFuA samples and the pCyDMA-*co*-THFuA fibre mat, suggesting that even though the window size of the fibres is larger than an individual *P. aeruginosa* cell, the change in surface architecture from flat to fibrous had no effect on the biofilm formation. In the case of *S. aureus* biofilm formation, this change in architecture had a small but not statistically significant effect on the fibres compared to the flat equivalent polymer. The difference in performance is possibly related to the differences in motile behaviour of the two pathogens. *P. aeruginosa* moves *via* swimming, swarming and twitching motility mechanisms whereas *S. aureus* is non-motile.^27^ Hence when *S. aureus* cells become lodged within the fibre material, they are less able to escape compared to the *P. aeruginosa*. This could inform future design where reducing the window size could lead to further reductions in *S. aureus* biofilm formation.^28,29^

When comparing the data from 2D samples, it was notable that they all exhibited significantly lower biofilm compared to the control glass sample. There was no significant difference for *P. aeruginosa* biofilm formation between the pCyDMA and CyDMA-*co*-THFuA sample that were coated onto coated glass coverslips, suggesting that the copolymerisation strategy had improved the mechanical properties without a loss of efficacy against this pathogen. However, a small but significant (*p* = 0.0303) increase was observed for *S. aureus* biofilm formation for the same comparison. The inclusion of THFuA was used successfully lower the *T*_g_ and allow for successful electrospinning manufacture which was not possible with CyDMA alone. THFuA also was used for its pro-healing properties including ability to increase fibroblast attachment and proliferation.^17^ Further optimization may identify a copolymer ratio that enables both effective electrospinning conditions and reduced *S. aureus* biofilm formation.

**Fig. 5:**
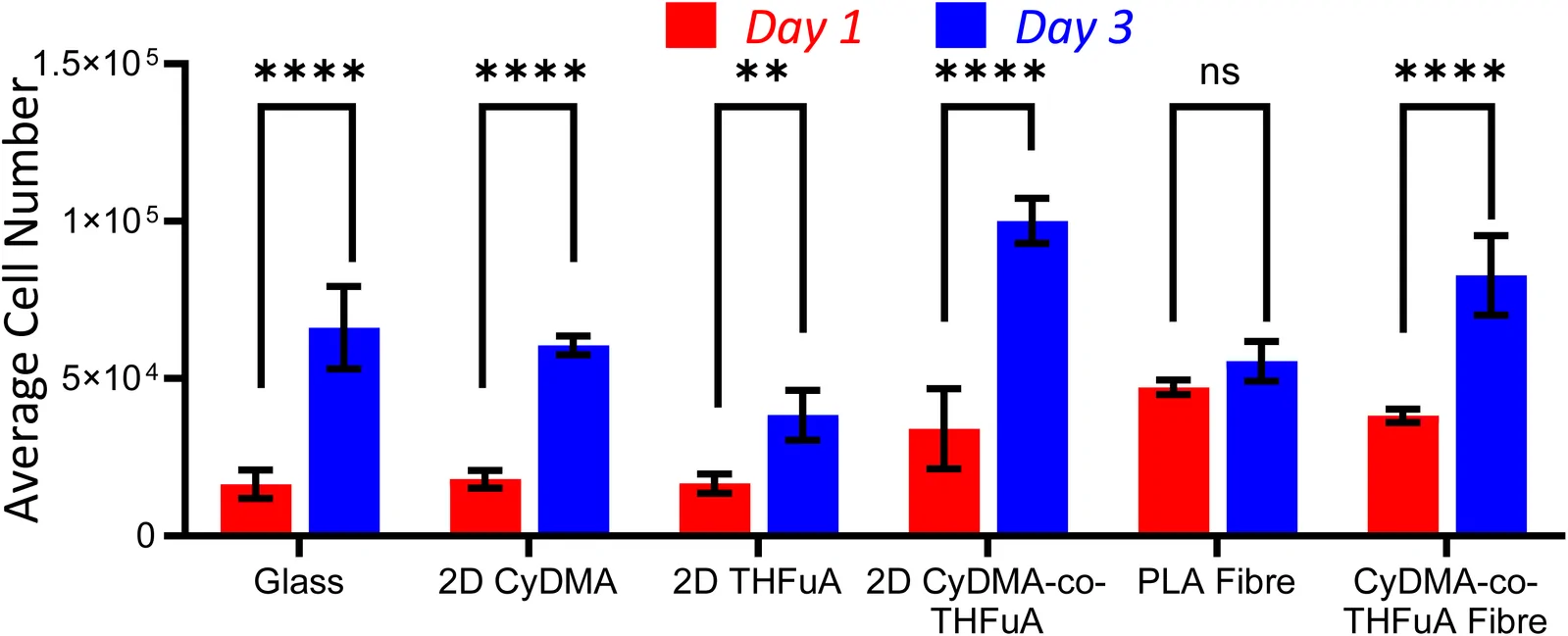
MTS assay data showing proliferation of BJ fibroblast cells on Glass, 2D CyDMA, 2D THFuA, 2D CyDMA-*co*-THFuA, PLA fibre and CyDMA-*co*-THFuA fibre samples. MTS assay was performed on Day 1 and Day 3. Error bars represent ± 1SD from *n* = 4 measurements. Statistical significance was calculated using one-way ANOVA where *p* values: * < 0.05, ** < 0.01, *** < 0.001 and **** < 0.0001 were used.

To assess the biocompatibility of the manufactured meshes, an MTS assay was used to observe cytotoxicity and proliferation of human dermal fibroblasts. It is acknowledged that the MTS assay is a measure of cell metabolism and therefore an indirect measure of cell number. Cell viability was assessed on days 1 and 3 of culture and compared to flat and fibre controls (Fig. 5). Initial attachment after 24 h on 2D samples showed that the CyDMA-*co*-THFuA (50 : 50) polymer promoted fibroblast attachment compared to other flat samples on average by a 2-fold increase and demonstrated a pro-proliferative surface with a higher cell number after 3 days.

This pro-proliferative nature was also demonstrated on the fibre samples. Initial attachment was shown to be higher on the PLA fibre sample where the PLA sample showed a small but statistically insignificant 1.2-fold increase in cell number from day 1 to 3 compared to a significant (*p* < 0.0001) increase of 2.2-fold for the pCyDMA-*co*-THFuA fibre sample. Surfaces with increased fibroblast proliferative effects have been shown to enhance wound healing due to the key role in forming granulation tissues.^30^ The combination of both anti-biofilm and pro-proliferative properties suggests that this fibre material could have great benefit in surgical meshes to both prevent infections and promote faster wound healing. Future work is warranted to assess more clinically relevant surgical site infection models such as polymicrobial and bacterial/immune cell assays both *in vitro* and *in vivo*. Additionally, to prove commercial viability, scale-up studies of the polymer and electrospinning processes will be used to demonstrate economic feasibility to generate the next generation of bio instructive meshes.

## Conclusions

A new bio-instructive fibre mat has been developed through the synthesis and electrospinning of a CyDMA and THFuA copolymer. This was found to successfully retain the properties of the two homopolymers; bacterial biofilm inhibition (CyDMA) and enhanced cell proliferation (THFuA). A series of co-polymer ratios were synthesised and the co-polymer ratio of (50 : 50), with *T*_g_ of 29 °C, was shown to be more processable *via* electrospinning to create scalable fibre mats with fibre diameter of 2.28 ± 0.65 µm. This pCyDMA-*co*-THFuA bio-instructive mat was then able to reduce the biofilm formation of *Pseudomonas aeruginosa* and *Staphylococcus aureus* by 84% and 59% respectively compared to an electrospun PLA fibre mat with similar fibre diameters. The bio-instructive pCyDMA-*co*-THFuA fibre mat was also shown to be able to increase fibroblasts proliferation by 2.2-fold over 3 days compared to only 1.2-fold observed in the PLA fibre mat sample. This study has shown how using two materials with different bio-instructive properties with unsuitable thermal properties for structurally resilient materials individually can be used to create a potential medical gauze with favourable thermal properties and synergistic bio-instructive properties. This approach has developed a material with potential to create hernia heshes that prevent infections in patients, to reduce the burden on the healthcare industry and to relieve patient suffering which could transform the quality of life for patients.

## Author contributions

All authors contributed to the interpretation of the data and to the review & editing of the manuscript. Joseph Sefton: writing – original draft, investigation. Michael P. Avery: writing – original draft, investigation. Jean-Frédéric Dubern: investigation. Mohammed Ghasemzadeh-Hasankolaei: investigation. Rahul Tiwari: resources, Amir M. Ghaemmaghami: resources, supervision. Morgan R. Alexander: conceptualization, funding acquisition, resources. Paul Williams: resources, supervision. Derek J. Irvine: conceptualization, funding acquisition, supervision. Jonny J. Blaker: conceptualization, funding acquisition, supervision. Adam A. Dundas: conceptualization, funding acquisition, investigation, writing – original draft, supervision.

## Conflicts of interest

There are no conflicts to declare

## Supplementary Material

LP-004-D5LP00220F-s001

## Data Availability

The data supporting the findings of this study are available in the University of Nottingham Data Repository at https://doi.org/10.17639/nott.7582. Supplementary information (SI) is available. See DOI: https://doi.org/10.1039/d5lp00220f.
