# Supplementary material for: Bioinstructive polymer fibre mats to reduce bacterial pathogen colonisation
Source: RSC Appl Polym. 2025 Nov 17;4(1):336–44. doi: 10.1039/d5lp00220f (PMC13592384; doi:10.1039/d5lp00220f)
Supplement: Supplementary file 1 [file LP-004-D5LP00220F-s001.pdf]

Supplementary Information (SI) for RSC Applied Polymers.
This journal is © The Royal Society of Chemistry 2025
Please do not adjust margins

SUPPLEMENTARY INFORMATION

Bioinstructive Polymer Fibre Mats to Reduce Bacterial Pathogen Colonisation

Joseph Sefton[a†], Michael P. Avery[b†], Jean-Frédéric Dubern[c], Mohammad Ghasemzadeh-Hasankolaei[d], Rahul Tiwari[d], Amir M Ghaemmaghami[d], Morgan R. Alexander[f], Paul Williams[c], Derek J. Irvine[a,g], Jonny J. Blaker[b,h], Adam A. Dundas[a*]

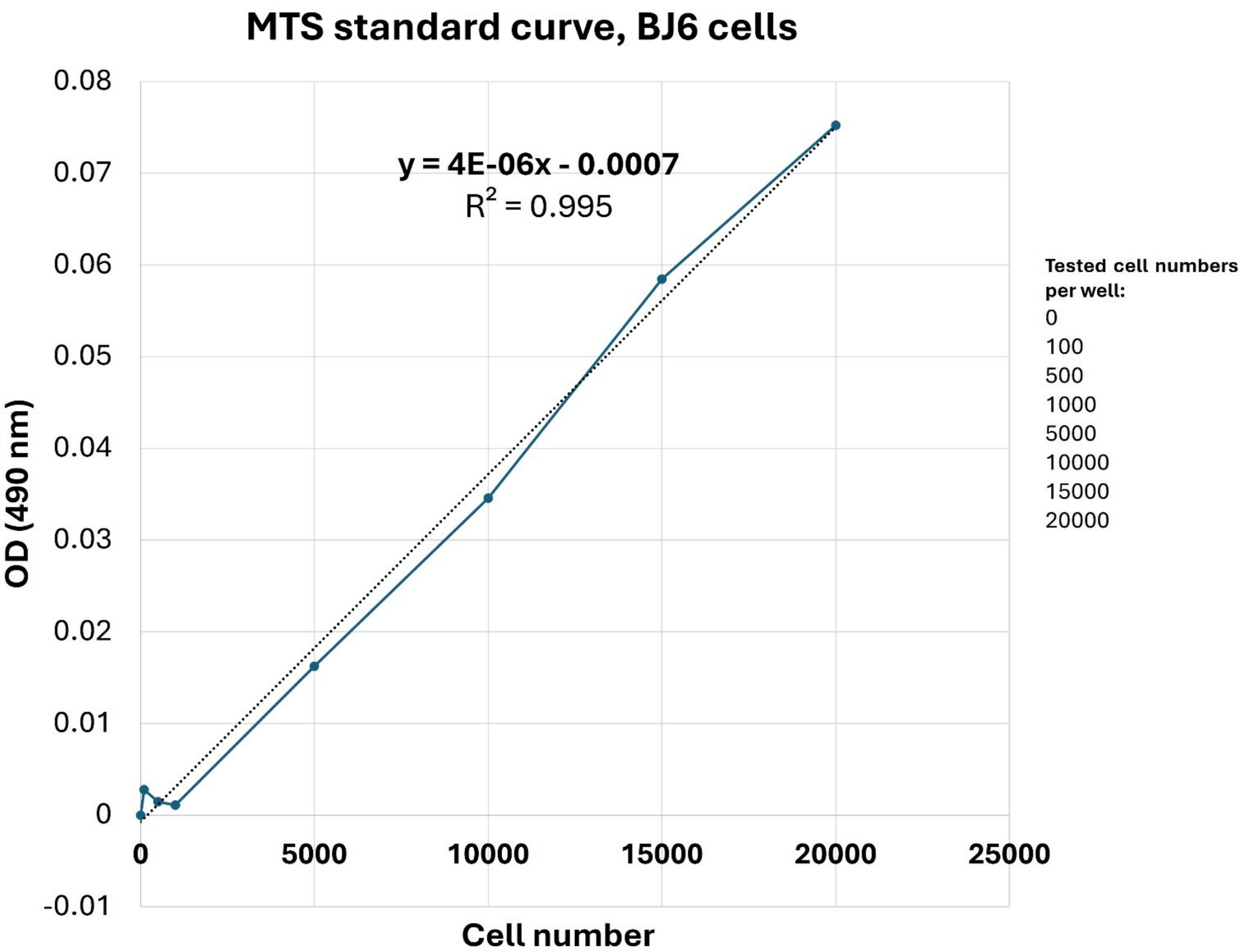

Figure S1: Standard curve for the MTS proliferation assay. Passage 7 cells seeded at 0, 100, 500, 1000, 5000, 10000, 15000 and 20000 cells per well in a 24 well plate. MTS reagent was added, and the optical density of aliquots were measured at 490 nm and the average used to plot the standard curve

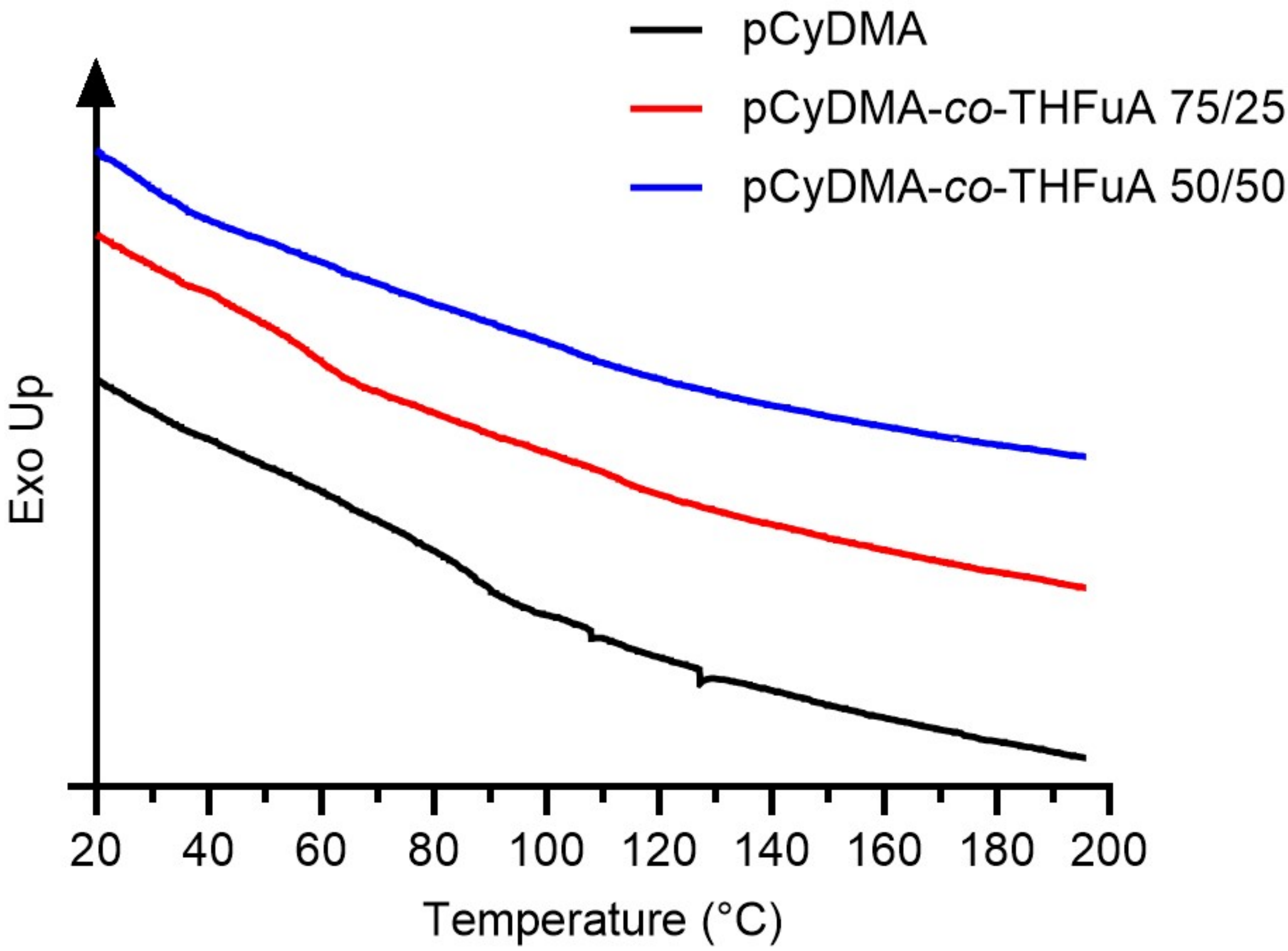

Figure S2: Differential scanning calorimetry results for pCyDMA, pCyDMA-*co*-THFuA 75/25 and pCyDMA-*co*-THFuA 50/50.

[a] *Department of Chemical and Environmental Engineering, Faculty of Engineering, University of Nottingham, Nottingham, NG7 2RD, UK*
[b] *Henry Royce Institute, The University of Manchester, Manchester, M13 9PL, UK*
[c] *National Biofilms Innovation Centre, Biodiscovery Institute and School of Life Sciences, University of Nottingham, NG7 2RD, UK*
[d] *School of Life Sciences, University of Nottingham, NG7 2RD, UK*
[e] *Camstent Ltd, The Exchange, Colworth Science Park, Bedford, MK44 1LZ*
[f] *Advanced Materials & Healthcare Technologies, School of Pharmacy, University of Nottingham, NG7 2RD, UK*
[g] *Centre for Additive Manufacturing, The University of Nottingham, Nottingham, NG7 2RD*
[h] *Department of Materials, The University of Manchester, Manchester, M13 9PL, UK*
† These authors contributed equally to the manuscript.
E-mail: adam.dundas1@nottingham.ac.uk
Supplementary Information available: [details of any supplementary information available should be included here]. See DOI: 10.1039/x0xx00000x

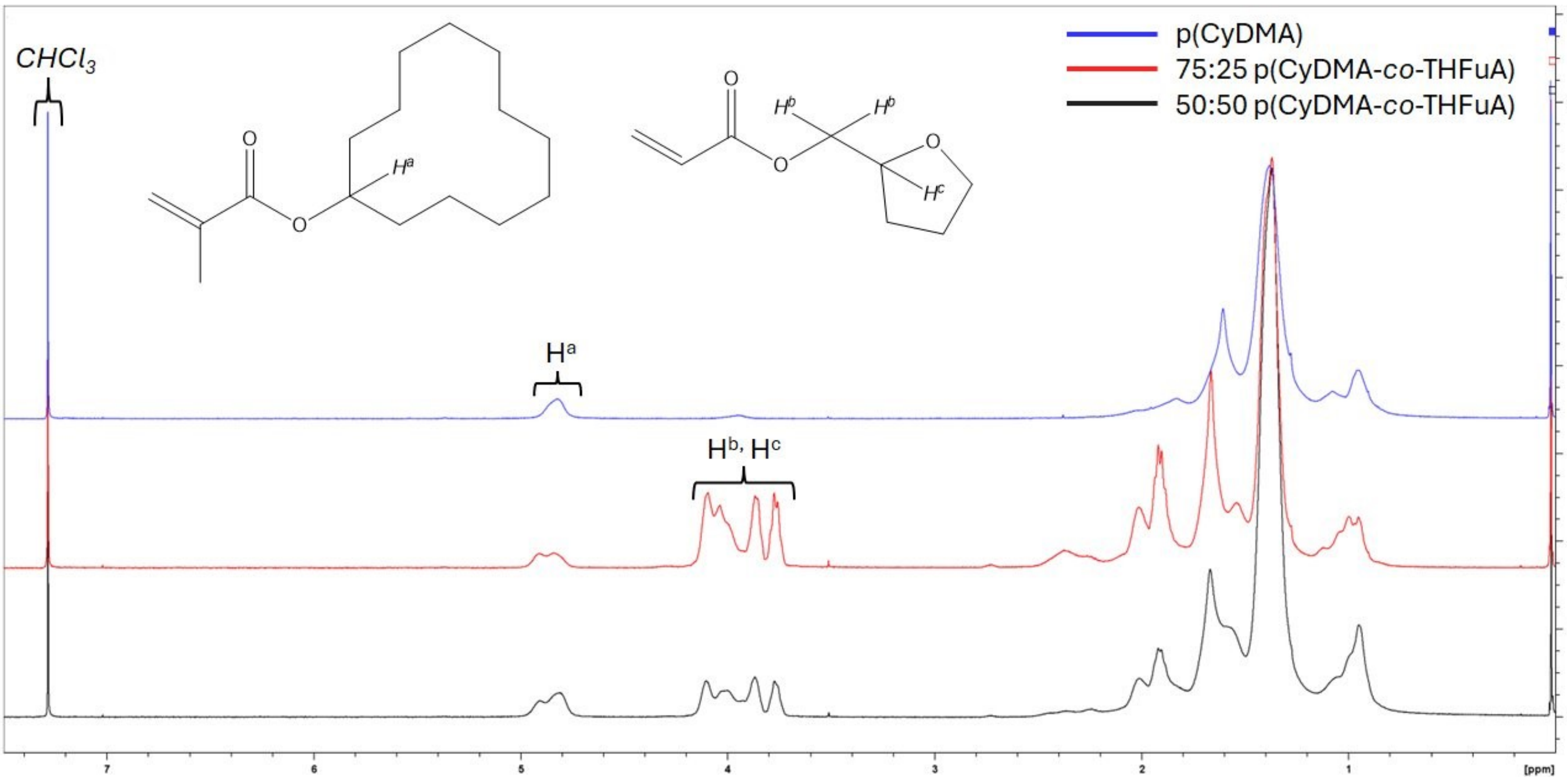

Figure S3: Stacked ^{1}H NMR spectra for p(CyDMA), 75:25 and 50:50 p(CyDMA-co-THFuA) showing the characteristic resonances of CyDMA and THFuA that were used for analysis

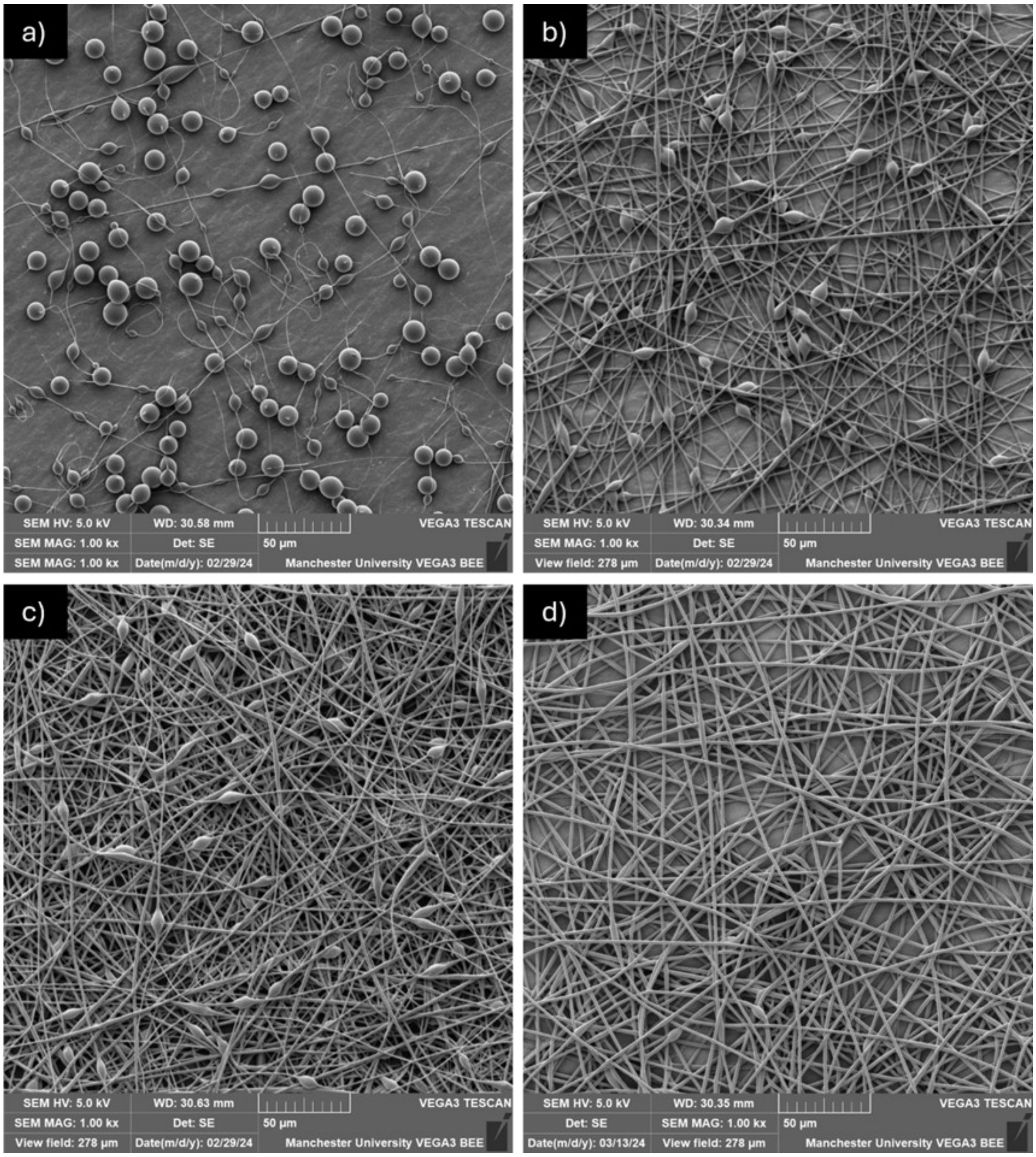

Figure S4: Scanning electron micrographs showing improvement in fibre formation from a 20% w/v solution of pCyDMA-*co*-THFuA (50:50) in a) DCM, b) 6.5 :1 DCM:EtOH, c) 4:1 DCM:EtOH, and d) 2:1 DCM:EtOH. All electrospinning experiments were performed with a –20 kV emitter voltage and a +1 kV collector voltage, solution flow rate of 3.5 ml h^{-1} with a working distance of 240 mm. All images are taken at 1000× magnification and scale bars represent 50 μm.

Table S1: Average fibre diameter of pCyDMA-*co*-THFuA 50:50 spun under the same conditions from varying DCM:EtOH solvent mixes as determined from the micrographs in Figure S3. Data from 100 fibres in a single image. For fibre measurements, bead diameters were not considered

DCM:EtOH	Average Fibre Diameter (µm)	Standard Deviation (µm)
1:0	N/A	N/A
6.5:1	1.28	0.35
4:1	1.36	0.36
2:1	1.88	0.24

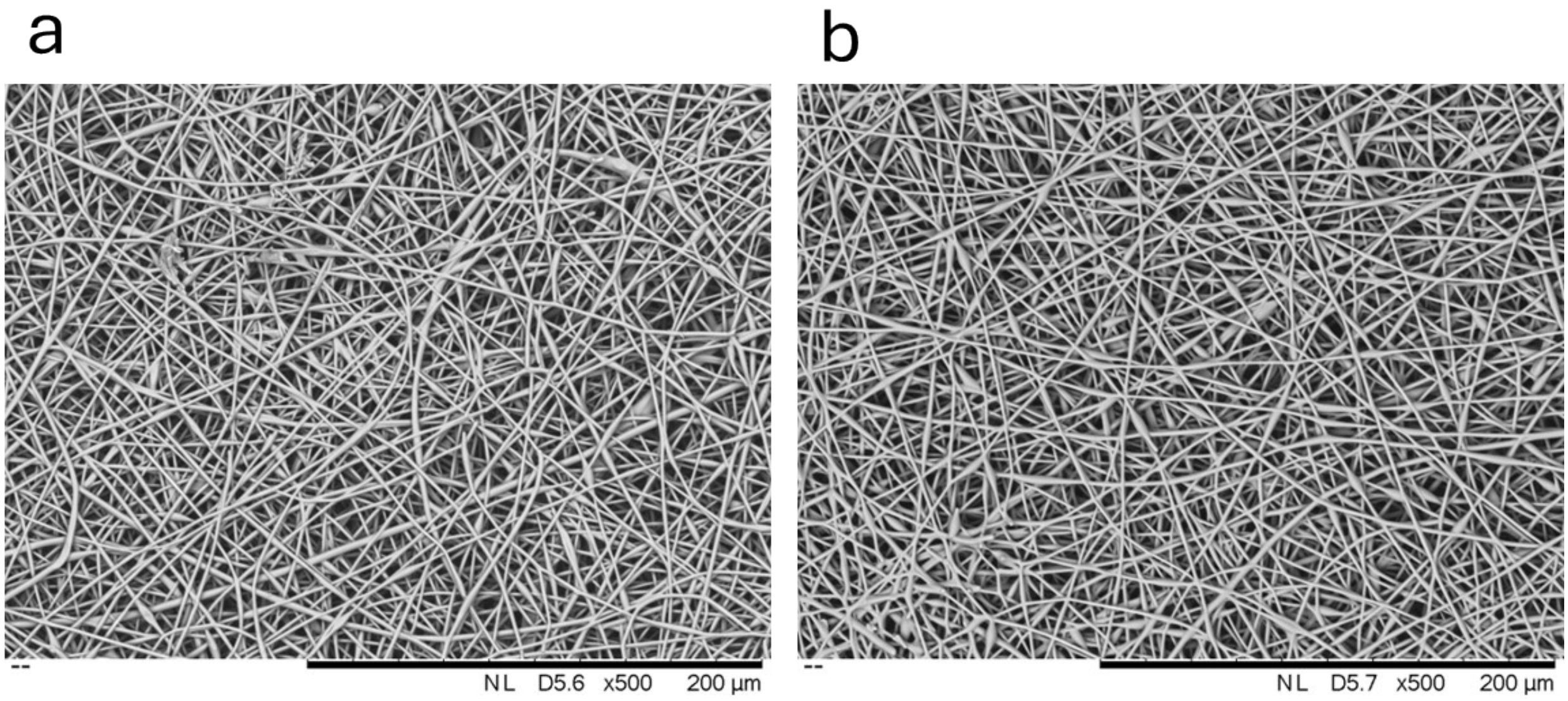

Figure S5: SEM images showing bio-instructive CyDMA-co-THFuA (50:50) polymer fibres (a) before gamma sterilisation and (b) after gamma sterilisation with a dose between 28.00 and 32.89 kGy.

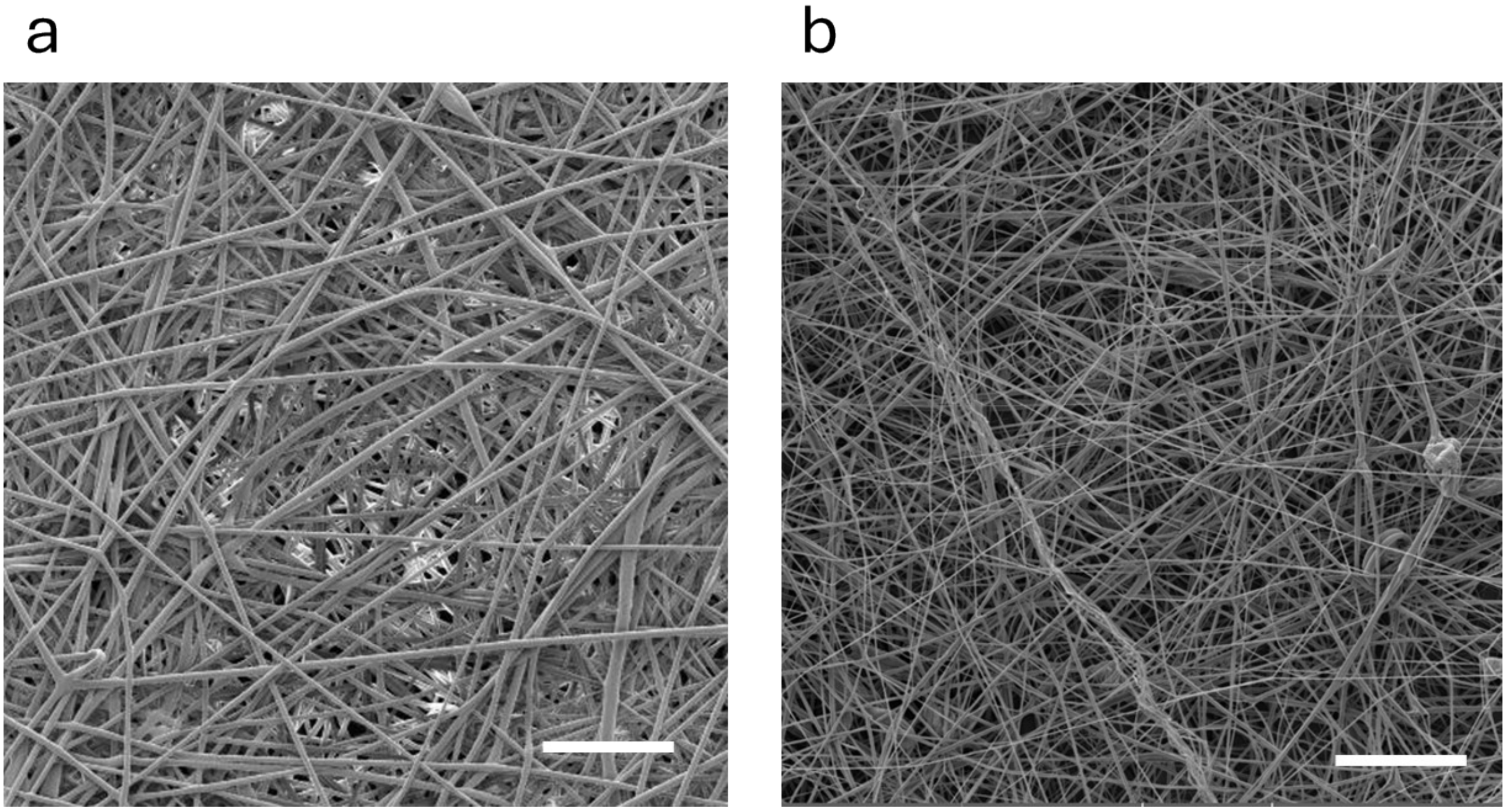

Figure S6: SEM images showing bio-instructive fibres used in biological studies (a) CyDMA-co-THFuA (50:50) 2.28 ± 0.65 μm (b) PLA fibres 1.20 ± 0.39 μm. Measurements were taken from N = 400 fibres. Scale bar represents 50 μm.
